# Vicarious Menstruation Following an Accident to the Finger

**Published:** 1844-05-04

**Authors:** 


VICARIOUS MENSTRUATION FOLLOWING AN ACCIDENT TO THE FINGER. REMOVAL OF THE FINGER, &C.

P. Lusanot, ætat. 17, a delicate young woman, of strumous diathesis, was admitted into the Royal Free Hospital, under the care of Mr. Gay, June 16th. In the month of August, 1842, she accidentally chopped off a portion of the indx-finger of the left hand. From a want of surgical interference the integuments retracted and closed around the bone, leaving its extremity exposed. She had not menstruated during the previous three years (the cause of the cessation of the menstrual function being unknown,) but in November the stump poured out, in the course of two or three days, a considerable quantity of dark fluid, resembling menstruous discharge, and continued to do so at intervals of a month, until the time of her admission. At the University College Hospital, where she had been for several weeks, the usual means were tried in order to effect a restoration of the uterine function, but without the least success. As the protruded bone appeared to be in a diseased and ulcerated condition, and the integuments towards the extremity of the stump had also an unhealthy aspect, Mr. Gay deemed it advisable to remove the remainder of the finger, which was accordingly done on the 22d of June, by cutting through the metacarpal bone. The usual discharge from the finger had just taken place, and the local symptoms of excitement which accompanied it had subsided, yet during the operation an unusual number of arteries bled freely, and several ligatures were required in order to stem the hæmorrhage. In the removed portion of the finger the bone was found to be spongy and very vascular, and the dorsal arteries large. The only impediment to the healing of the wound was the occurrence of slight hæmorrhage, of a venous character, on the second day after the operation, which was checked by the use of cold water. One ounce of the compound steel mixture was directed to be given three times a day, and an aloetic pill, with the use of the hip bath, each night.

On the 15th of July, up to which time the patient had gone on well, she complained of pain from the shoulder to the hand, with a sense of throbbing along the course of the large vessels, and the head begun to swell. A dozen leeches were applied to the pubic region, and a brisk aloetic purgative given to her; but the pain continued, the radial and ulnar arteries pulsated vigorously, the extremity became hot, the fingers rigidly flexed, and the hand fearfully distended, the integuments assuming a dark-bluish tinge. On the following day another dozen of leeches were applied to the legs, and saline aperient medicine substituted for the steel mixture, until the excitement shall have passed away. Under this treatment the symptoms began to subside, and by the 31st the hand and arm had regained their usual size, and their vessels resumed their healthy action. The steel mixture was now resumed, with the addition of fifteen grains of ergot of rye to each dose; and the palm of the hand having been carefully padded with cotton wool, and laid with the fingers on a hand-splint, the extremity was swathed with narrow bandages from the tips of the fingers and thumb to the shoulder.

On the 16th of August, after an interval of freedom from pain, it returned in the shoulder, and the symptoms of the previous month began to show themselves. Dry cupping was immediately performed over the loins, and injections of warm milk, with the strong solution of ammonia, in the proportion of a drachm and a half of the latter to a pint, were ordered to be thrown into the vagina night and morning. The pains continued until the 20th, but no swelling took place; this appeared to be prevented by the bandages, which were kept accurately and constantly applied, and the period passed away without any other disturbance.

On the 30th the bandages were removed, the arm and hand became œdematous in the course of a few hours, but the œdema subsided on the renewed application of the bandages.

In September the pains were felt in the arm. On the 11th and on the 14th the patient suffered severely from the tension of the whole limb, which she said was so great that she feared it would burst the bandages asunder. She was dry-cupped again on the loins, her strength not being such as to justify the abstraction of blood. The pain became now transferred to the back of the neck, to which part the cupping-glasses were applied on the 15th, with signal relief, and from this period the symptoms again declined. The injections produced a sensation of heat and prickling in the os uteri and over the pubic region, but, together with the usual emmengogue remedies proved inadequate to restore the uterine functions.

The young woman now wished to go into the country, and left the hospital for that purpose, promising to continue the use of the bandages, and to return if any untoward symptom should show itself; since that period she has not visited the institution.

The treatment pursued in the foregoing case was suggested by the conviction that if a metastasis of the effort, which in the diseased finger appeared to supersede normal menstruation, could be effected to the uterus, its function would be restored; but that towards bringing about such a metastasis the usual emmenagogue remedies would be of little or no avail unless their employment was preceded by a removal of the diseased finger-stump, which not only furnished an opportune channel for the vicarious discharge, but was also a powerful agent in diverting the menstrual effort from its appropriate organ. There could not be discovered, from examinations which were made, any physical causes why the uterus should have suspended its menstrual functions; nor why the employment of the various remedies which were used did not prove successful in restoring it, especially since it was obvious that the menstrual effort, or "vis," was in a condition of periodical activity in the system. Probably the change of air and other circumstances may in due time do for the young woman what the contents of the materia medica appear to be incapable of effecting. —*London Lancet.*

